# Abnormally Elevated Blood Tacrolimus Level Following the Concomitant Use of Nirmatrelvir/Ritonavir With Extended-Release Tacrolimus in a Post-lung Transplant Patient: A Case Report and a Literature Review

**DOI:** 10.7759/cureus.62868

**Published:** 2024-06-21

**Authors:** Hikari Yoshida, Takumi Umemura, Soichiro Ito, Takahito Mizuno, Yoshikazu Mutoh, Tetsuya Yamada, Tomoki Kimura

**Affiliations:** 1 Department of Pharmacy, Tosei General Hospital, Seto, JPN; 2 Department of Infectious Diseases, Tosei General Hospital, Seto, JPN; 3 Department of Respiratory Medicine and Allergy, Tosei General Hospital, Seto, JPN

**Keywords:** nirmatrelvir/ritonavir, tacrolimus, drug interaction, covid-19, lung transplant

## Abstract

Although nirmatrelvir/ritonavir (NMV/r) reportedly increases blood levels of tacrolimus (TAC) due to CYP3A4 inhibition and other factors, reports on the use of NMV/r in combination with tacrolimus hydrate extended-release capsules (TAC-ER) in lung transplant patients are limited. Herein, we present a case with post-lung transplantation of elevated blood trough levels of TAC after concomitant use of NMV/r. A woman in her 60s had undergone lung transplantation. She had coronavirus disease 2019 (COVID-19) and was co-administered NMV/r and TAC-ER, with the trough level controlled at approximately 4 μg/mL. Upon the co-administration of NMV/r and TAC-ER, the patient developed diarrhea and vomiting and was hospitalized. TAC-ER was discontinued on day 6, and TAC level was measured on day 8 and had risen above 100 ng/mL. This level gradually decreased to 17.8 ng/mL on day 11 and 2.4 ng/mL on day 15; therefore, TAC-ER was resumed at 2.5 mg/day. On day 18, the TAC level was 5.2 ng/mL, which was within the target range, and the patient was discharged on day 19. This is the first report of a post-lung transplant patient co-administered TAC-ER with NMV/r, who showed abnormally high blood TAC levels above the detection limit. In patients using TAC-ER after lung transplantation, it may be useful to confirm that the TAC blood level is below the effective therapeutic range before resuming TAC-ER safely.

## Introduction

Patients receiving solid organ (e.g., kidney and lung) transplants are at a high risk of severe coronavirus disease 2019 (COVID-19) caused by severe acute respiratory syndrome coronavirus 2 (SARS-CoV-2), which is associated with a high mortality rate [1]. Nirmatrelvir/ritonavir (NMV/r) is used as an oral treatment for mild or moderate COVID-19 to reduce the risks of hospitalization and death [2,3]. Ritonavir is a pharmacokinetic (PK) enhancer that can prolong the half-life (t1/2) of nirmatrelvir mainly by inhibiting cytochrome P450 (CYP) 3A4-mediated metabolism of nirmatrelvir. Similarly, ritonavir inhibits CYP3A4, and to a lesser extent, CYP2D6 and P-glycoprotein (P-gp); therefore, the interactions of ritonavir with other drugs should be considered [4,5]. Tacrolimus (TAC), which has a narrow therapeutic concentration range, necessitating close monitoring to prevent rejection and avoid toxicity, is used post-lung transplantation [6,7]. Monitoring blood levels of TAC is important because elevated blood levels of TAC may have adverse events such as kidney injury [8]. Although NMV/r reportedly increases the blood levels of TAC due to CYP3A4 inhibition and other factors, reports on the use of NMV/r in combination with tacrolimus hydrate extended-release capsules (TAC-ER) in lung transplant patients are limited [9]. We encountered a post-lung transplant patient on TAC-ER who had COVID-19 and elevated TAC blood levels after the concomitant use of NMV/r.

## Case presentation

A woman in her 60s had hypoparathyroidism, human T-lymphotropic virus type I-associated myelitis, and chronic kidney disease. The patient had been taking TAC-ER at 2.5 mg/day to prevent lung allograft rejection, and the trough concentration was controlled at approximately 4 μg/mL. The blood TAC was measured by a chemiluminescent enzyme immunoassay using an ARCHITECT i1000SR analyzer (Abbott Diagnostics, North Chicago, IL, USA). She had no particular allergies, but had a history of bezafibrate-induced kidney injury. She was hospitalized for fever and cough with sputum. Simultaneously, we tested for SARS-CoV-2 antigen, and the test was positive during the omicron wave in April 2022. She was diagnosed with bronchiectasis after recovering from a pulmonary non-tuberculous mycobacterial infection and had undergone bilateral lung transplantation eight years ago.

Her regular medications comprised TAC-ER 2.5 mg (two capsules of 1.0 mg and one capsule of 0.5 mg, after breakfast), bisoprolol fumarate 2.5 mg (1.5 tablet, after breakfast), amlodipine besilate 5 mg (one oral dissolve tablet, after breakfast), rabeprazole sodium 10 mg (one tablet, after breakfast), prednisolone 5 mg (two tablets, after breakfast), mycophenolate mofetil 250 mg (one capsule each, BID, after breakfast and dinner), polaprezinc 75 mg (one tablet each, BID, after breakfast and dinner), calcium polystyrenesulfonate 20% 25 g (one pack, after lunch), atovaquone 15% 750 mg (one pack, after breakfast), pantothenic acid 100 mg (one tablet each, BID, after breakfast and dinner), magnesium oxide 330 mg (one tablet each, BID, after breakfast and dinner), eprazinone hydrochloride 20 mg (one tablet each, TID, after meal), tranexamic acid 250 mg (one capsule each, TID, after meal), L-carbocisteine 500 mg (one capsule each, TID, after meal), Bacillus butyrate (one tablet each, TID, after meal), pregabalin 25 mg (one oral dissolve each, BID, after breakfast and dinner), and alfacalcidol 1 μg (one capsule, after breakfast).

On day 1 of her illness, her vital signs were as follows: temperature, 37.5°C; respiratory rate, 20 breaths per min; and oxygen saturation, 98%. She was diagnosed with mild COVID-19 according to the World Health Organization Criteria [10]. On the same day, the patient was prescribed NMV/r (nirmatrelvir 300 mg, ritonavir 100 mg, BID for five days) against the estimated glomerular filtration rate (eGFR) of 28.56 mL/min. Subsequently, the patient was advised to rest and be treated at home. None of the drugs was discontinued during the initiation of NMV/r, and all drugs were continued at the same dose. Although there were concerns about the interactions between NMV/r and TAC-ER, TAC-ER was continued considering the chances of rejection in the chronic phase after transplantation.

**Figure 1:**
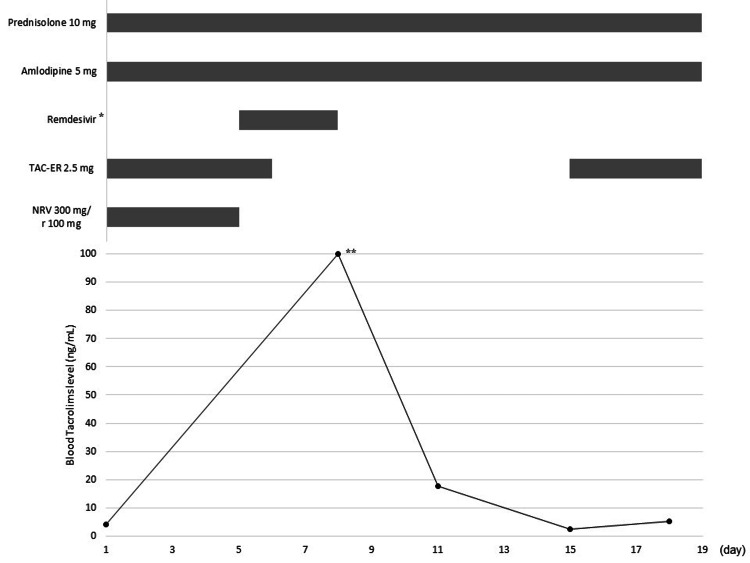
TAC blood concentration trends and the TAC-ER and NMV/r dosing course TAC-ER: tacrolimus hydrate extended-release capsules; NMV/r: nirmatrelvir/ritonavir * 200 mg on the first day and 100 mg after the second day, ** >100 above

On day 3 of her illness, the patient developed diarrhea and vomiting, and stopped taking pantothenic acid and magnesium oxide. However, her symptoms did not improve, and she was hospitalized on day 5. On admission, her vital signs were as follows: temperature, 36.8°C; respiratory rate, 18 breaths per min; and oxygen saturation, 98%. Diarrhea and vomiting were thought to be caused by NMV/r; therefore, NMV/r was discontinued, and remdesivir and sotrovimab were administered. TAC-ER and other drugs were discontinued on day 6 and TAC level was measured on day 8. The TAC level had risen above 100 ng/mL; furthermore, her serum potassium level was high at 5.8 mmol/L. No significant changes were observed in serum creatinine levels - 1.16 mg/dL on day 1 and 1.17 mg/dL on day 8. The TAC level gradually decreased to 17.8 ng/mL on day 11 and 2.4 ng/mL on day 15; therefore, TAC-ER was resumed at 2.5 mg/day. On day 18, the TAC level was 5.2 ng/mL, which was within the target range, and the patient was discharged on day 19. Figure 1 shows the TAC blood concentration trends and TAC-ER and NMV/r dosing course.

## Discussion

This is the first report of a post-lung transplant patient co-administered TAC-ER with NMV/r who showed abnormally high blood TAC levels above the detection limit. It has been reported that the blood TAC level may increase abnormally owing to increased bioavailability caused by inhibition of CYP3A4 and P-gp in the small intestine and decreased clearance caused by inhibition of CYP3A4 in the liver by NMV/r [11]. In particular, ritonavir is known to be a potent inhibitor of CYP3A4, the main metabolizing enzyme of TAC, and appears to be a major factor in the elevated blood TAC level in this case. Although it has been reported [12] that diarrhea increases the blood trough concentration of TAC, this might not have been a major factor in this case, because the reported blood TAC level was 11.3 ng/mL, a negligible increase compared with that in our case. It is known that the expression of P-gp, for which TAC is a substrate, is inhibited by nirmatrelvir [13]. However, it has been reported [14] that the effect of the concomitantly administered NMV on the blood level of dabigatran, which reportedly increases due to the inhibition of P-gp expression, is negligible. It can be inferred that the effect of P-gp inhibition by NMV on the increase in blood TAC level was also negligible in our case; however, further studies are needed in this regard. The mechanism of CYP3A4 inhibition by ritonavir, which has a significant effect on the increase in blood TAC level, is irreversible, and takes several days for this inhibitory effect to disappear [9]. After day 8, when the CYP3A4-inhibitory effect of ritonavir disappeared, the blood TAC level decreased according to its t1/2 [15]. Medications co-administered with NMV/r, such as amlodipine and prednisolone, reportedly increase blood TAC levels, however, none of these medications had stronger interactions than NMV/r [16-18]. In addition, blood TAC levels reportedly increased upon remdesivir administration, which is used to treat patients with COVID-19 after NMV/r discontinuation [19]. However, remdesivir limitedly affects blood TAC levels because it is rapidly excreted [16]. Thus, none of these medications would present potent interactions. Although it is possible that concomitant medications other than NMV/r, such as amlodipine, prednisolone, and remdesivir, may have had an effect on the increase in blood TAC levels, we considered that none of these medications had potent interactions.

We reviewed the literature on organ transplant patients using TAC-ER in combination with NMV/r. Table 1 presents the background of post-solid organ transplant patients using TAC-ER in combination with NMV/r and Table 2 presents the blood TAC level course of these patients [20-23]. All patients using TAC-ER, except for the present patient, were post-kidney transplant patients. In four of six patients using TAC-ER (present case, and reported cases (RCs) 1, 3, and 4), the blood TAC levels after the resumption of TAC-ER increased compared with the level before NMV/r initiation. In lung transplant patients, TAC blood levels are controlled at 8-12 ng/mL three months after transplantation and at 5-15 ng/mL in renal transplant patients [24]. Herein, it was controlled at approximately 4 ng/mL because kidney injury occurred when she was administered TAC-IR; however, rejection did not occur even when the TAC blood level was controlled to be low. TAC is metabolized in the liver, and because the RCs who used TAC-ER in renal transplantation were many years post-renal transplantation as in the present case; thus, it is unlikely that hepatopathy-induced PK changes will occur in the acute phase after transplantation. Therefore, there may be no significant difference in the PK of TAC between kidney and lung transplant patients. Many cases reported abnormally high TAC blood levels after renal transplantation (RC21), but in the RC, as in our case, we did not believe that discontinuing TAC after initiating NMV/r was the cause. Therefore, if TAC monitoring is performed to confirm that the concentration drops within the normal range and TAC-ER is restarted, it may be possible to prevent deviations in TAC concentration.

**Table 1:** List of reported organ transplant patients using tacrolimus hydrate extended-release capsules with nirmatrelvir/ritonavir RC: reported case; TAC: tacrolimus; NMV/r: nirmatrelvir/ritonavir

	Our Case	RC1 [20]	RC2 [21]	RC3 [22]	RC4 [23]	RC5 [23]
Male(M)/female(F)	F	F	F	F	M	F
Age (y)	60s	80s	80s	30s	70s	50s
Years since transplantation (y)	8	9	13	16	4	6
Replacement organ	lung	kidney	kidney	kidney	kidney	kidney
TAC maintenance dosage (mg/day)	2.5	5	5	8	1.5	5
Blood TAC level before the start of NMV/r (ng/mL)	4.0	5.2	7.9	7.5	6.6	10.5
NMV dosage (mg/day)	300	unknown	300	300	unknown	unknown
Day of resumption of TAC from NMV/r started (d)	15	6	11	8	9	9
TAC dosage of restarted (mg/day)	2.5	5	1	4	1.5	5
Ratio of TAC dose (resumption/maintenance) (%)	100	100	20	50	100	100

**Table 2:** Changes in tacrolimus concentration after taking nirmatrelvir/ritonavir in patients reporting tacrolimus hydrate extended-release capsules use * TAC-ER discontinuation All reported cases used NMV/r on days 1-5. Our case used NMV/r on days 1-4. RC: reported case; TAC-ER: tacrolimus hydrate extended-release capsules; NMV/r: nirmatrelvir/ritonavir

Day	Tacrolimus levels (ng/mL)																							
	1	2	3	4	5	6	7	8	9	10	11	12	13	14	15	16	17	18	19	20	21	22	23	24
Our Case	-	-	-	-	-	*-	*-	*>100	*-	*-	*17.8	*-	*-	*-	*2.4	-	-	5.2	-	-	-	-	-	4.4
RC1 [20]	*-	*4.5	*-	*4.7	*-	*-	-	16.2	-	-	-	-	-	-	-	-	-	-	-	-	-	-	-	-
RC2 [21]	-	-	-	*-	*-	*112	*-	*-	*27.6	*14.1	*8.2	-	5.7	-	-	6.2	-	-	-	-	-	-	-	-
RC3 [22]	*-	*-	*10	*-	*11	*-	*8	*7	11	11.5	-	-	-	6	-	-	-	5.5	-	-	-	-	-	-
RC4 [23]	*-	*-	*8.6	*-	*-	*6.8	*-	*-	*5.6	-	-	-	-	7.8	-	-	-	-	-	-	-	-	-	-
RC5 [23]	*-	*10.5	*-	*9.1	*-	*-	*-	*-	*<2.0	-	-	-	-	-	-	-	7.2	-	-	-	-	-	-	-

Table 3 shows the background of the post-lung transplant patients who received TAC in combination with NMV/r and Table 4 shows the blood TAC level course of these patients [25,26]. The post-lung transplant patients were using TAC-immediate release (IR) except our patient. The maximum blood level of a single 1.5 mg dose of TAC is 2.287 ng/mL for TAC-ER and 7.331 ng/mL for TAC-IR, which differ in the absorption process, but have similar t1/2 of 36.1 and 37.2 h [15], respectively. Therefore, it is considered that differences in dosage forms do not have a significant effect on t1/2. However, RC18 resumed the 100% dose of TAC-IR, but it was discontinued again owing to high TAC blood levels. No high levels were observed in our case, even when TAC-ER was resumed at 100% dose. TAC-IR and TAC-ER are administered at different times in stable lung transplant patients. If the total daily dose is identical, the 24-h AUC and trough levels do not differ significantly, however, the trough levels tend to be slightly higher [27]. Therefore, although patients being administered TAC-IR, may have resumed TAC-IR too early after NMV/r completion, TAC trough levels may have been lower in patients administered TAC-ER than in those administered TAC-IR. However, frequent TAC blood measurements are necessary in all cases. The increase in blood TAC levels after the resumption of TAC-ER may have been minor in our case because NMV/r was discontinued for four days and the duration of irreversible inhibition of CYP3A4 by ritonavir was shorter than that if the patient had completed five days of treatment. The blood TAC levels may have increased further after TAC-ER resumption if NMV/r had not been discontinued on day 4. In addition, the blood TAC levels were elevated in all four patients who resumed treatment at the same dose. Furthermore, there have been reports of an increase in the blood TAC level compared to the baseline blood TAC level even when TAC was resumed at a reduced dose, regardless of the TAC formulation or transplanted organ. Recommending a uniform date to resume TAC is difficult, considering this case and the 18 RCs, as the increased TAC levels in the blood due to NMV/r are affected by factors such as the date on which TAC was discontinued. Therefore, the timing of TAC resumption should be carefully determined. However, in post-transplant patients, as in our patient, there is a risk of rejection after chronic lung transplantation because of a drop in blood levels after TAC-ER discontinuation. If discontinuing TAC is difficult because of the potential for such interactions, switching to other antivirals (such as remdesivir or molnupiravir), which have relatively fewer interactions with TAC, may be an option.

**Table 3:** List of reported lung transplant patients with nirmatrelvir/ritonavir and tacrolimus RC: reported case; TAC: tacrolimus; NMV/r: nirmatrelvir/ritonavir; ER: extended-release; IR: immediate-release

	Our Case	RC6 [25]		RC7 [25]	RC8 [25]	RC9 [25]	RC10 [25]	RC11 [25]	RC12 [25]	RC13 [25]	RC14 [25]	RC15 [25]	RC16 [25]	RC17 [25]	RC18 [26]
Male(M)/female(F)	F	F	F		M	F	M	M	F	M	M	M	F	M	M
Age (y)	60s	60s	30s		50s	30s	60s	70s	30s	50s	50s	60s	50s	40s	40s
Years since transplantation (y)	8	1	2		1	3	12	2	12	0.25	5	3	13	4	8
TAC maintenance dosage (mg/day)	2.5	3.5	4		4	7	1.5	6.5	3.5	2.5	1.5	2	2	3	1
Blood TAC level before the start of NMV/r (ng/mL)	4.0	14.5	10.9		7.7	8.2	5.2	8.9	5.0	9.7	4.5	5.8	5.8	6.4	10.0
TAC formulation	ER	IR	IR		IR	IR	IR	IR	IR	IR	IR	IR	IR	IR	IR
NMV dosage (mg/day)	300	600	600		600	600	600	600	600	600	600	600	600	600	600
Day of resumption of TAC from NMV/r started (d)	15	8	8		8	8	8	8	8	8	8	8	8	7	6
TAC dosage of restarted (mg/day)	2.5	0.5	1		1	2	0.5	2	1	0.5	0.5	0.5	2	1	1
Ratio of TAC dose (resumption/maintenance) (%)	100	15	25		25	29	33	31	29	20	33	25	100	33	100

**Table 4:** Changes in tacrolimus concentration after taking nirmatrelvir/ritonavir in patients reporting lung transplantation * TAC-ER discontinuation All reported cases used NMV/r on days 1-5. Our case used NMV/r on days 1-4. RC: reported case; TAC-ER: tacrolimus hydrate extended-release capsules; NMV/r: nirmatrelvir/ritonavir

Day	TAC levels (ng/mL)																								
	1	2	3	4	5	6	7	8	9	10	11	12	13	14	15	16	17	18	19	20	21	22	23	24	25
Our Case	-	-	-	-	-	*-	*-	*>100	*-	*-	*17.8	*-	*-	*-	*2.4	-	-	5.2	-	-	-	-	-	-	4.4
RC6 [25]	*-	*-	*-	*6.2	*-	*5.5	*-	*-	-	4.1	-	4.8	-	-	-	-	11.2	-	-	-	-	-	-	-	-
RC7 [25]	*-	*-	*-	*-	*9.7	*-	*-	*7.8	-	-	-	8.7	-	-	8.2	-	-	-	-	-	-	-	-	-	-
RC8 [25]	*-	*-	*-	*11	*-	*-	*-	*-	-	-	8	-	-	-	-	-	-	-	-	-	-	-	-	-	-
RC9 [25]	*-	*-	*-	*6.5	*-	*-	*-	*-	-	-	-	-	-	-	-	-	-	-	-	-	-	-	-	-	-
RC10 [25]	*-	*-	*-	*6.7	*-	*-	*-	*-	-	-	4.3	-	-	-	-	-	-	-	-	-	-	-	-	-	8.6
RC11 [25]	*-	*-	*-	*-	*6.4	*-	*-	*-	-	-	-	10.3	-	-	-	-	-	-	-	-	-	-	-	-	-
RC12 [25]	*-	*-	*-	*-	*-	*-	*-	*-	-	7.9	-	-	-	-	-	-	-	-	-	-	-	-	-	-	-
RC13 [25]	*-	*-	*16.8	*5.3	*-	*-	*-	*-	-	16.5	-	-	-	7.8	-	-	-	-	-	-	-	-	-	-	-
RC14 [25]	*-	*-	*-	*4.4	*-	*-	*-	*-	-	-	8.2	-	-	-	-	-	-	-	-	-	-	-	-	-	-
RC15 [25]	*-	*-	*-	*-	*7.2	*-	*-	*-	-	-	-	12.5	-	-	-	-	-	-	-	-	-	-	-	-	-
RC16 [25]	*-	*-	*-	*6.5	*-	*-	*-	*-	-	-	10.6	-	-	-	-	4.8	-	-	-	-	-	-	-	-	-
RC17 [25]	*-	*-	*-	*8	*-	*-	*-	-	-	5.9	-	-	-	-	-	-	-	-	-	-	-	-	-	-	-
RC18 [26]	*-	*-	*-	*-	*-	*-	-	-	-	-	-	*31.6	*-	*16	*-	*-	-	-	9.6	-	-	-	-	-	-

## Conclusions

In patients using TAC-ER after lung transplantation, it may be useful to confirm that the blood TAC levels are below the effective therapeutic range when the TAC concentration is measured before safely resuming TAC-ER.
